# The Phagocytosis of *Lacticaseibacillus casei* and Its Immunomodulatory Properties on Human Monocyte-Derived Dendritic Cells Depend on the Expression of Lc-p75, a Bacterial Peptidoglycan Hydrolase

**DOI:** 10.3390/ijms23147620

**Published:** 2022-07-10

**Authors:** Márta Tóth, Szabolcs Muzsai, Krzysztof Regulski, Tímea Szendi-Szatmári, Zsolt Czimmerer, Éva Rajnavölgyi, Marie-Pierre Chapot-Chartier, Attila Bácsi

**Affiliations:** 1 Department of Immunology, Faculty of Medicine, University of Debrecen, 4032 Debrecen, Hungary; toth.marta@med.unideb.hu (M.T.); muzsai.szabolcs@med.unideb.hu (S.M.); rajnavolgyi.eva@gmail.com (É.R.); 2 Doctoral School of Molecular Cell and Immune Biology, University of Debrecen, 4032 Debrecen, Hungary; 3 ELKH-DE Allergology Research Group, 4032 Debrecen, Hungary; 4 Gyula Petrányi Doctoral School of Clinical Immunology and Allergology, University of Debrecen, 4032 Debrecen, Hungary; 5 INRAE, AgroParisTech, Micalis Institute, Université Paris-Saclay, 78350 Jouy-en-Josas, France; krzysztof.regulski@cellectis.com (K.R.); marie-pierre.chapot-chartier@inrae.fr (M.-P.C.-C.); 6 Department of Biophysics and Cell Biology, Faculty of Medicine, University of Debrecen, 4032 Debrecen, Hungary; szatmari.timea@med.unideb.hu; 7 Department of Biochemistry and Molecular Biology, Faculty of Medicine, University of Debrecen, 4032 Debrecen, Hungary; czimmerer.zsolt@med.unideb.hu

**Keywords:** lactobacillus, dendritic cells, phagocytosis, immunomodulation

## Abstract

The human gut symbiont *Lacticaseibacillus (L.) casei* (previously *Lactobacillus casei*) is under intense research due to its wide range of immunomodulatory effects on the human host. Dendritic cells (DCs) are crucial players in the direct and indirect communication with lactobacilli in the gastrointestinal tract. Here, we demonstrate that human monocyte-derived DCs (moDCs) are able to engulf *L. casei* BL23, in which the intact bacterial cell wall and morphology have a key role. The absence of the bacterial cell-wall-degrading enzyme, Lc-p75, in *L. casei* cells causes remarkable morphological changes, which have important consequences in the phagocytosis of *L. casei* by moDCs. Our results showed that the Lc-p75 mutation induced defective internalization and impaired proinflammatory and T-cell-polarizing cytokine secretion by bacteria-exposed moDCs. The T helper (Th) 1 and Th17 cell activating capacity of moDCs induced by the mutant *L. casei* was consequently reduced. Moreover, inhibition of the phagocytosis of wild-type bacteria showed similar results. Taken together, these data suggested that formation of short bacterial chains helps to exert the potent immunomodulatory properties of *L. casei* BL23.

## 1. Introduction

Lactobacilli are Gram-positive lactic acid bacteria (LAB) that are natural inhabitants of the human gastrointestinal tract. They are also widely used in fermented food production, and certain strains are commercialized as probiotics with health-promoting properties. Probiotics are “live microorganisms that, when administered in adequate amounts, confer a health benefit on the host” [1]. A wide range of beneficial effects is attributed to probiotics, including maintaining gut epithelial barrier integrity [2], preventing the adhesion of pathogenic bacteria [3,4], and secreting vitamins [5] and metabolites [6]. Furthermore, probiotics contribute to the development of the immune system and modulate immune responses [7,8,9].

Bacteria must directly or indirectly interact with both nonimmune and immune cells of the host to exert their beneficial effects. Considering the unique and complex functional activities of dendritic cells (DCs) in modulating both innate and adaptive immunity, they became first-line candidates for characterizing the strain-specific actions of various probiotic bacteria, such as lactobacilli. DCs express unique sets of surface-bound and intracellular pattern recognition receptors (PRRs), which sense various microbe-associated molecular patterns (MAMPs) [10]. As professional phagocytes, DCs can internalize microbes upon recognition of MAMPs. This results in the activation of signaling cascades, after which DCs produce soluble factors that shape the innate and adaptive immune responses; further, as professional antigen-presenting cells, DCs present peptide antigens to naïve T cells, mediating their activation and polarization [11].

Bacterial peptidoglycan (PG) molecules play an essential role in bacterial life and host–microbe interactions. The synthesis and degradation of PG are tightly regulated by bacterial enzymes, including peptidoglycan hydrolases (PGHs), which can cleave the PG macromolecule at well-defined sites [12]. It has previously been reported that p40 and p75 enzymes are PGHs with pleiotropic effects on bacterial cells. The two proteins are localized in the bacterial cell wall and are released to the environment freely [13] or bound to extracellular vesicles [14]. It has been demonstrated that these enzymes play a role in *L. casei* growth and cell division [13,15]. Interestingly, the *L. casei* p40 (Lc-p40) and p75 (Lc-p75) mutant strains remarkably differ in their morphology. Lacking Lc-p40 causes only minor changes; bacteria assume a curved shape, while the absence of Lc-p75 leads to the formation of a long-chain, auto-aggregation phenotype [13,15]. Moreover, Lc-p75 is endowed with γ-d-glutamyl-l-lysyl-endopeptidase activity, and its inactivation causes altered composition of PG fragments [15] that serve as MAMPs and modulate the host’s immune responses. Bacterial PG, a major structural cell wall component that originates from pathogens [16] or probiotic [17] bacteria, induces DC activation. However, the impacts of bacterial PG alterations on DC activation and functions are less studied.

In this study, we aimed to explore the DC-modulatory properties of *L. casei* BL23 and its ∆Lc-p75 mutant derivative. With the help of the chain-forming ∆Lc-p75 mutant, we showed that phagocytosis was a critical element in the modulation of DC functions by the probiotic bacteria. We observed that impaired phagocytosis of the mutant bacteria by human monocyte-derived DCs (moDCs) altered their T-cell polarizing ability. These observations may contribute to a deeper understanding of the modulatory mechanisms exerted by probiotic bacteria. Furthermore, they might help generate probiotic bacterial strains, which can have remarkable therapeutic potential in different diseases via the modulation of immune responses.

## 2. Results

### 2.1. Lc-p75 Deficiency Does Not Influence the moDC-Activating Potential of L. casei, but Affects the Inflammatory Cytokine Secretion by Bacteria-Exposed moDCs

**Figure 1:**
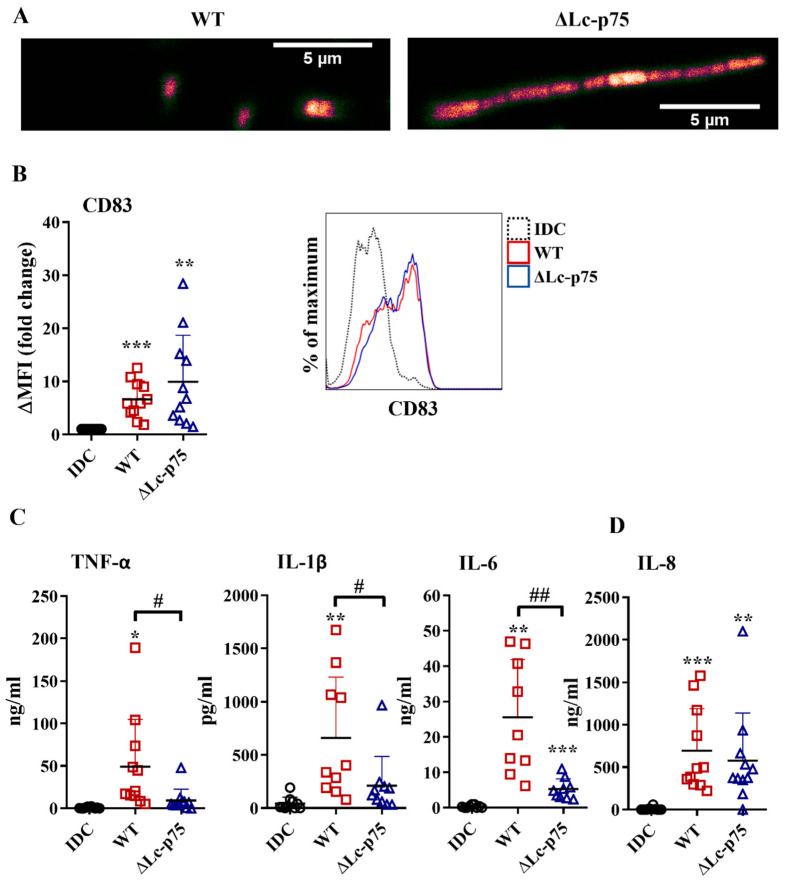
The absence of Lc-p75 PGH affects bacterial morphology and the inflammatory mediators’ production, but not the CD83 expression by bacteria-activated moDCs. (**A**) Confocal microscopic pictures of mCherry-expressing wild-type (WT) and ΔLc-p75 *L. casei* strains. Five-day moDCs were co-incubated with WT and PGH mutant bacteria at a ratio of 1:4 for 24 h. The expression of CD83 (**B**) was measured by flow cytometry. Fold change of median fluorescent intensities (ΔMFI) compared to the control, nontreated cells (immature DC/IDC/) was calculated from 6–9 independent experiments ±SD. Histogram overlays show one representative experiment. Each dot is representative of one donor. The concentrations of proinflammatory TNF-α, IL-1β, and IL-6 cytokines (**C**) and IL-8 chemokine (**D**) were measured using ELISA from the supernatant of activated moDCs and control, nonactivated cells (IDC). Figures show the mean of 9–11 independent experiments ±SD. Statistical analysis was performed using the Student’s paired two-tailed *t*-test with significance defined as * *p* < 0.05, ** *p* < 0.01, and *** *p* < 0.001 as compared to IDC. The differences between WT and ΔLc-p75 were statistically significant and defined as # *p* < 0.05 and ## *p* < 0.01.

First, we investigated the effects of wild-type (WT) *L. casei* and its mutant counterpart, which was defective in Lc-p75 cell wall-degrading enzyme, on moDC activation. The ΔLc-p75 mutant was obtained via deletion of the gene encoding the major PGH in *L. casei* BL23 [15]. As expected, the ΔLc-p75 mutant strain had a modified cell wall structure and morphology compared to the WT *L. casei* strain, as shown by confocal microscopy (Figure 1A).

To assess the impact of Lc-p75 inactivation on the inflammatory-provoking capacity of moDCs, we studied the CD83 expression and inflammatory cytokine and chemokine secretion by moDCs treated with WT and ΔLc-p75 *L. casei* strains for 24 h at a ratio of 1:4. Membrane-bound CD83 is strongly expressed by mature, gut-bacteria activated moDCs [18,19]. In our experimental system, *L. casei* strains also increased the expression level of CD83; however, no statistically significant difference was observed between WT and mutant strains with respect to the degree of CD83 elevation (Figure 1B). In contrast, WT bacteria induced a significantly higher amount of TNF-α, IL-1β, and IL-6 cytokines than their defective counterpart (Figure 1C). Interestingly, we did not detect any significant differences in IL-8 (Figure 1D) chemokine secretion by moDCs activated with the WT or PGH mutant bacteria. To exclude that the differences in moDC activating abilities of the WT and ΔLc-p75 *L. casei* strains were the consequence of the different levels of treatment-induced cell death, we measured the frequency of dead cells in bacteria-exposed moDC cultures using flow cytometry. Remarkably, we could detect only an insignificant proportion of dead cells in moDC cultures incubated with either WT or mutant bacterial strains, similar to that observed in unstimulated moDC cultures (Figure S1).

These results collectively indicated that the *L. casei* mutant with a defective cell wall structure and altered morphology promoted an attenuated inflammatory response by moDCs. In contrast, the appearance of the activation marker CD83 on bacteria-exposed moDCs was not sensitive to the lack of bacterial Lc-p75 PGH.

### 2.2. Lc-p75-Deficiency in L. casei Does Not Influence the Antigen Presentation and Costimulation, but Induces Less T-Cell-Polarizing Cytokine Secretion by Bacteria-Exposed moDCs

**Figure 2:**
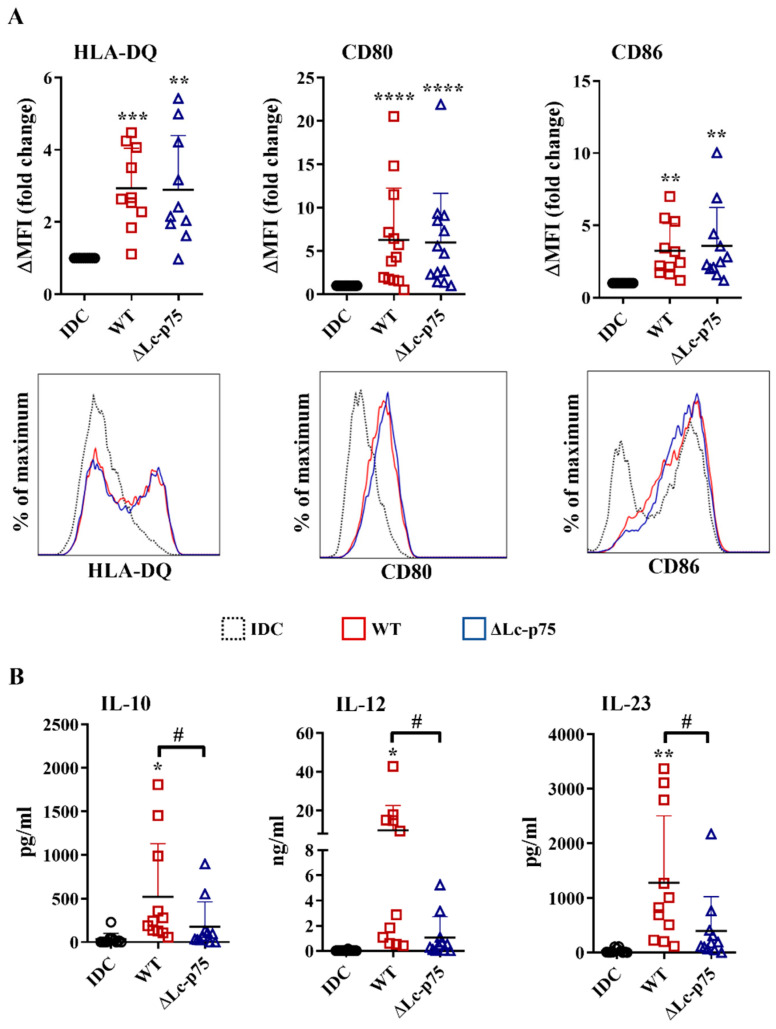
Deletion of Lc-p75 PGH in *L. casei* BL23 does not affect the antigen-presenting and costimulatory capacity of bacteria-exposed moDCs but causes decreased T-cell-polarizing cytokine secretion by these cells. Five-day moDCs were stimulated with live wild-type (WT) *L. casei* BL23 and ΔLc-p75 mutant strain at a ratio of 1:4 for 24 h. The expression of HLA-DQ, as well as costimulatory CD80 and CD86 molecules (**A**), was measured using flow cytometry. The fold change of the median fluorescent intensities (ΔMFI) compared to the control cells (IDC) was calculated from 10–13 independent experiments ±SD. Histogram overlays show one representative experiment. The concentrations of T-cell-polarizing IL-10, IL-12, and IL-23 cytokines (**B**) were measured using ELISA from the supernatant of activated moDCs and control, nontreated cells (IDC). Figures show the mean of 10–11 independent experiments ±SD. Each dot represents one donor. Student’s paired two-tailed *t*-test was used in the statistical analysis, with significance defined as * *p* < 0.05, ** *p* < 0.01, *** *p* < 0.001 and **** p < 0.0001 compared to IDC; and # *p* < 0.05 between WT and ΔLc-p75.

After recognizing pathogens, the highly phagocytic, immature DCs transform into a cell type that has the characteristics required for the activation and polarization of Th cells in different directions. One of the unique features of dendritic cells is the migration to the secondary lymphoid organs to activate naïve Th cells and provide costimulation simultaneously with the antigen presentation via MHCII molecules [20]. Therefore, an increased level of HLA-DQ, CD80, and CD86 co-stimulatory molecules is considered a good marker of stimulated DCs that are capable of T cell activation. After a 24 h incubation, the WT *L. casei* and ∆Lc-p75 mutant derivative induced the upregulation of HLA-DQ, CD80, and CD86 expression of moDCs, regardless of the mutation (Figure 2A).

In addition to inflammatory cytokine and chemokine secretion, DCs also secrete soluble mediators to shape the differentiation of various T cell subsets. Therefore, we measured the T-cell-polarizing IL-10, IL-12, and IL-23 production by moDCs after a 24 h activation with *L. casei* strains. Importantly, WT *L. casei* induced more robust IL-10, IL-12, and IL-23 secretion by moDCs than their PGH defective counterpart (Figure 2B).

These results indicated that the ∆Lc-p75 mutant *L. casei* strain caused dampened T-cell-polarizing cytokine secretion by moDCs, and consequently may have influenced their T-cell activating capacity. In contrast, expression of antigen-presenting and costimulatory molecules on bacteria-exposed moDCs did not depend on the presence of the bacterial Lc-p75 enzyme.

### 2.3. Activation and Polarization of Autologous T Lymphocytes by Bacteria-Exposed moDCs Depend on the Presence of Lc-p75 in L. casei BL23

**Figure 3:**
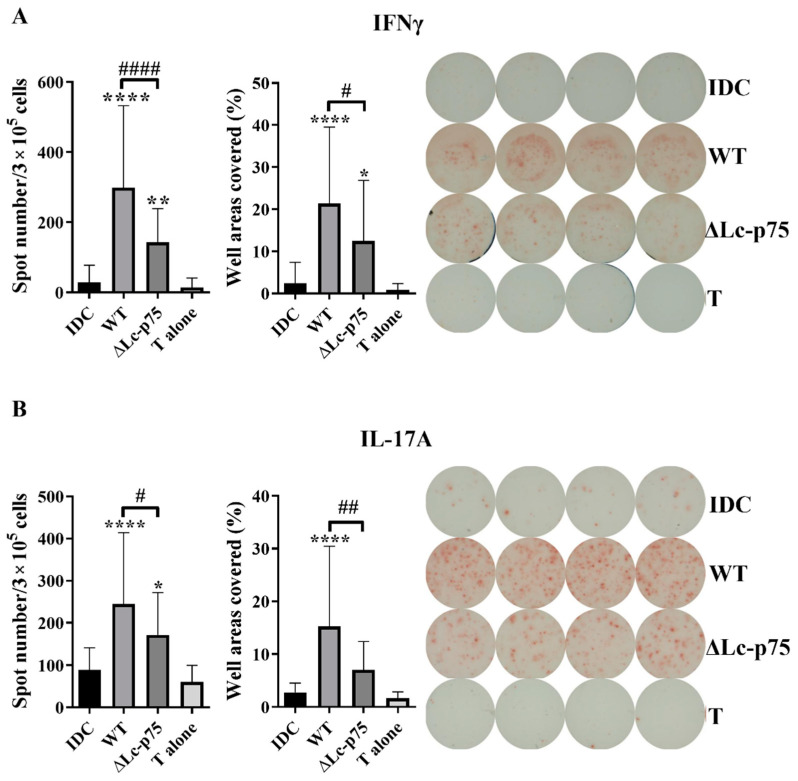
The T-cell-polarizing potential of moDCs is modulated by Lc-p75 inactivation in *L. casei* BL23. Five-day moDCs activated with wild-type (WT) and ∆Lc-p75 mutant *L. casei* BL23 were washed and then cocultured with autologous T cells from freshly isolated PBLs at a ratio of 1:20 for 3 days. The frequency of cytokine-producing T lymphocytes was measured using IFN-γ (**A**) or IL-17A (**B**) ELISPOT assays. The spot number was counted, or the area covered by the spots was calculated using a computer-assisted ELISPOT image analyzer. The mean value of spot numbers and well areas covered were calculated from 5 independent experiments with 4–6 parallel wells ±SD. One-way ANOVA followed by Tukey’s multiple-comparison test was used for statistical analysis. Significance was defined as * *p* < 0.05, ** *p* < 0.01 and **** *p* < 0.0001 compared to control, non-treated cells (IDC). The differences between WT and ΔLc-p75 were statistically significant and defined as # *p* < 0.05, ## *p* < 0.01, and #### *p* < 0.0001.

To study whether the decreased T-cell-polarizing cytokine secretion could influence the outcome of T lymphocyte responses mediated by the moDCs, we cocultured bacteria-activated moDCs with autologous peripheral blood lymphocytes (PBLs) for 3 days. T cell responses were monitored using IFNγ-, IL-17-, and IL-4-specific ELISPOT assays. Unstimulated moDCs and PBLs were used as negative controls. Higher numbers and a larger area of the spots were observed in cases of IFN-γ- and IL-17-producing T cells when the moDCs were prestimulated with *L. casei* strains compared to unstimulated moDC or PBL controls. Preactivation of moDCs with the *L. casei* ∆Lc-p75 mutant resulted in significantly lower numbers and a smaller area of spots in the case of IFN-γ-secreting T cells compared to pretreatment with WT bacteria (Figure 3A). Similarly, a significantly lower number of IL-17A-secreting T cells and less intense IL-17A production was induced by moDCs preactivated with ∆Lc-p75 mutant bacteria compared to pretreatment with WT bacteria (Figure 3B). In contrast, prestimulation of moDCs with *L. casei* strains resulted in a significant reduction in the spot numbers and the area coverage representing IL-4 production compared to control, untreated moDCs, independently of the PG mutation (Figure S2).

These results indicated that the Lc-p75 defective *L. casei* induced reduced Th1 and Th17 responses compared to WT *L. casei* BL23.

### 2.4. Peptidoglycan Derived from ∆Lc-p75 Mutant or WT L. casei BL23 Exhibits Similar moDC-Activating Potential

**Figure 4:**
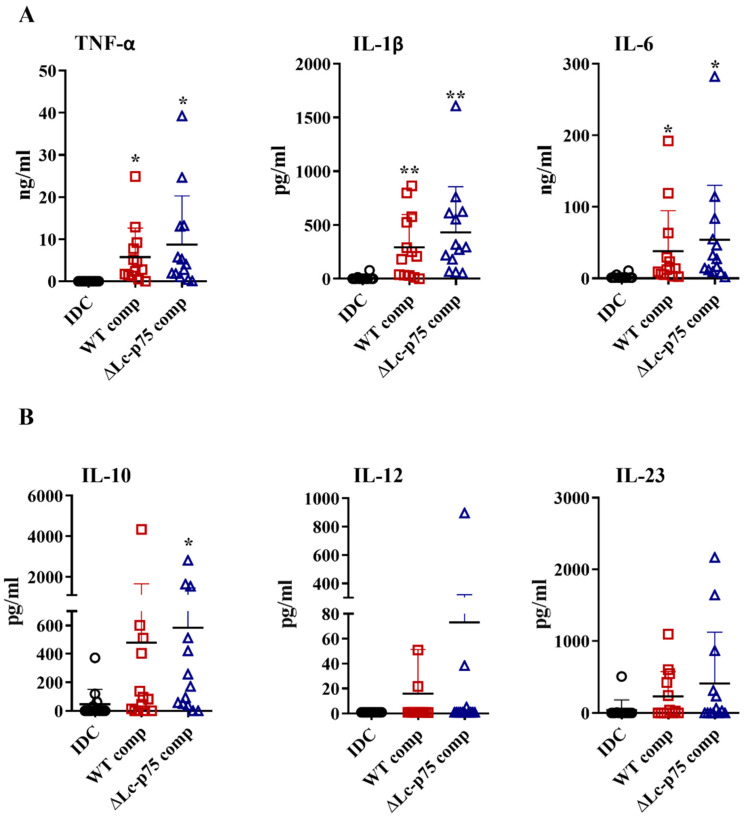
PG derived from wild-type (WT) *L. casei* BL23 and ∆Lc-p75 mutant strains induces enhanced cytokine secretion by moDCs independently of the PGH mutation. Five-day moDCs were treated with 10 μg/mL PG components derived from WT and Lc-p75 mutant strains for 24 h. The concentrations of TNF-α, IL-1β, and IL-6 proinflammatory cytokines (**A**) and of IL-10, IL-12, and IL-23 T-cell-polarizing cytokines (**B**) were determined using ELISA. The mean of the concentrations was calculated from 11–13 independent experiments. Each dot represents one donor. Student’s paired two-tailed *t*-test was used in the statistical analysis, with significance defined as * *p* < 0.05 and ** *p* < 0.01 compared to the control, nonactivated cells (IDC).

Based on the results above, we raised the question whether the differences between the WT and mutant *L. casei* strains in moDC activation were the direct consequences of the modified bacterial cell wall structure or linked indirectly to the altered bacterial morphology. Therefore, we purified PGs derived from the WT and ∆Lc-p75 mutant strains and tested their moDC-stimulating activity. PG preparations induced elevated proinflammatory (Figure 4A) and T-cell-polarizing (Figure 4B) cytokine secretion by moDCs independently of the targeted PGH mutation. Furthermore, PGs from both *L. casei* strains caused a significant elevation in CD83, HLA-DQ, CD80, and CD86 expression (Figure S3A), as well as in IL-8 production (Figure S3B). Importantly, these changes were not dependent on the targeted PGH mutation, similar to the results obtained with the live bacteria.

Taken together, these results demonstrated that the purified PGs from WT and mutant *L. casei* BL23 strains did not show any significant differences in their DC activating potentials.

### 2.5. Altered Bacterial Morphology Leads to Impaired Phagocytosis by moDCs

Since we could not detect any differences in moDC activation between WT and ∆Lc-p75 mutant *L. casei*-derived purified PGs, we hypothesized that the attenuated DC stimulation induced by the ∆Lc-p75 mutant *L. casei* BL23 was the consequence of their impaired phagocytic uptake by moDCs.

For the phagocytosis assay, in vitro differentiated 5-day moDCs were coincubated with bacteria of each strain expressing the mCherry fluorescent protein for 3 or 24 h. Within both incubation periods at 37 °C, moDCs were shown to internalize fluorescent bacteria, consequently becoming mCherry-positive. The percentage of these mCherry-positive phagocytic cells could be quantified by flow cytometry and was compared to control samples incubated on ice or in the absence of bacteria.

**Figure 5:**
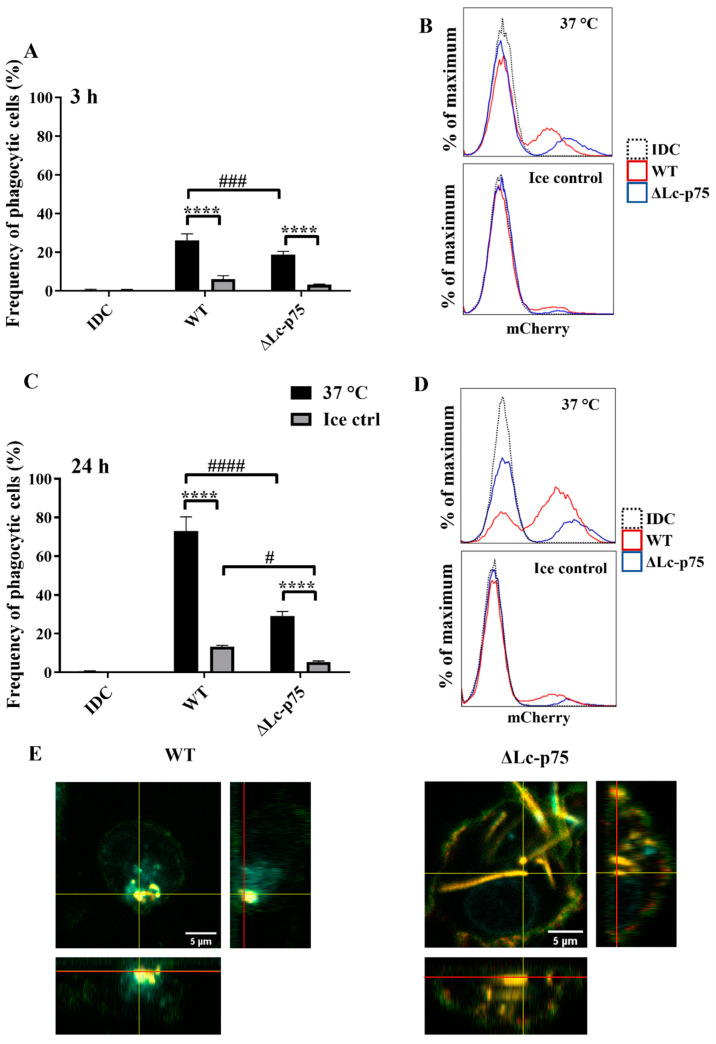
Inactivation of Lc-p75 in *L. casei* BL23 alters the phagocytic efficiency of moDCs. In vitro differentiated five-day moDCs were coincubated with mCherry-labeled WT *L. casei* BL23 and ∆Lc-p75 mutant for 3 and 24 h at a ratio of 1:4 at 37 °C and on ice as a control. Phagocytic activity of moDCs was measured by detecting mCherry fluorescent protein-positive moDCs with flow cytometry after 3 h (**A**,**B**) and after 24 h (**C**,**D**). IDC means nonactivated moDCs. Histogram overlays show the results of one representative experiment. On the flow cytometric figures, the mean values of 3 independent experiments are presented ±SD. For confocal microscopic pictures (**E**), moDCs are labeled with HLA-DQ (green), CD1a (red), and DAPI (cyan); mCherry is indicated with yellow. Orthogonal views are demonstrated for one Z-stack image. On the bottom is Z-projection in the X–Z direction, on the right is the Z-projection in the Y–Z direction. The red lines indicate the Z-depth of the optical slice and the orthogonal planes of the X–Z and Y–Z projections, respectively. Two-way ANOVA followed by Tukey’s multiple-comparison tests were used in the statistical analysis. Significance was defined as **** *p* < 0.0001 showing the differences between the samples incubated at 37 °C and on ice. The differences between WT and ΔLc-p75 were statistically significant and defined as # *p* < 0.05, ### *p* < 0.001, and #### *p* < 0.0001.

We found that WT bacteria were internalized with higher frequency by moDCs than mutant bacteria after 3 (Figure 5A,B) and 24 (Figure 5C,D) hours. The control samples incubated on ice exhibited a low percentage of mCherry-positive cells. The significantly higher frequency of mCherry-positive moDCs coincubated with WT *L. casei* BL23 at 37 °C indicated active, temperature-dependent internalization of bacteria within a 3 h incubation period (Figure 5A,B) and after 24 h of coincubation (Figure 5C,D). We confirmed the moDC-mediated uptake of WT and ΔLc-p75 mutant (Figure 5E) bacteria using confocal microscopy displaying orthogonal views of the Z-stack images.

**Figure 6:**
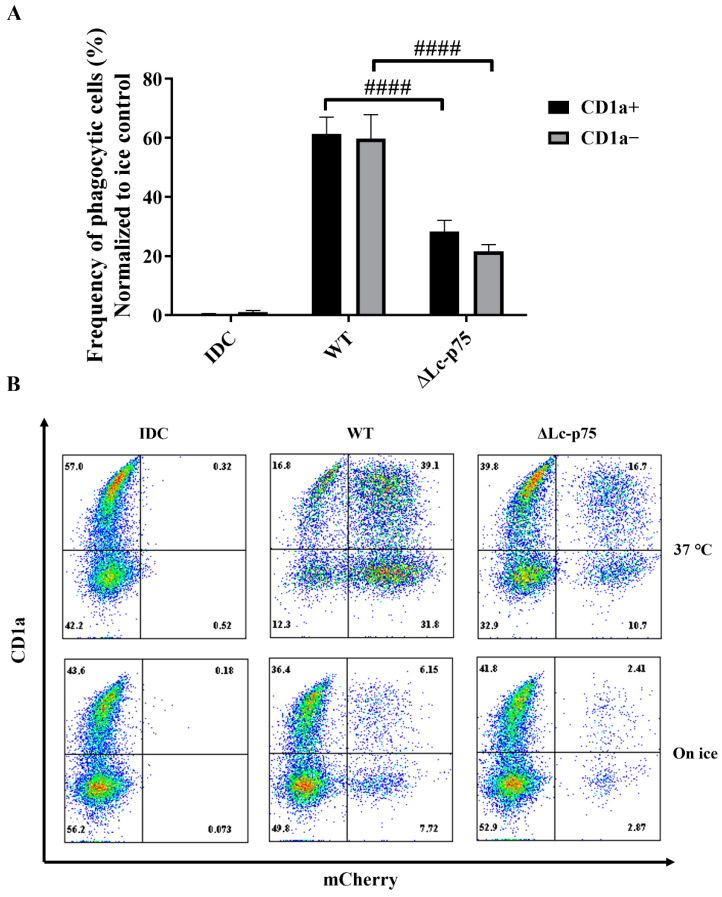
Phagocytic efficiency of moDCs activated with *L. casei* bacteria is independent of the CD1a expression. Five-day moDCs were stimulated with mCherry-labeled WT *L. casei* BL23 and ∆Lc-p75 mutant bacteria for 24 h at 37 °C and on ice. The phagocytic capacity of CD1a^+^ and CD1a^−^ subsets was measured by detecting mCherry-positive moDCs with flow cytometry (**A**). IDC represents the sample without any bacterial treatment. The frequency of phagocytic cells was normalized to ice control, and the mean values of 3 independent experiments are presented ±SD. (**B**) Dot plots from one experiment showing the CD1a and mCherry expression of moDCs. Two-way ANOVA followed by Tukey’s multiple-comparison tests were used in the statistical analysis. Differences between WT and ΔLc-p75 were statistically significant, and defined as #### *p* < 0.0001.

It has previously been reported that the CD1a^+^ and CD1a^−^ subsets of moDCs generated in vitro differed in their *Escherichia coli*-internalizing capacity [21]. However, in another study, Seshadri et al. demonstrated that the absence of the CD1a protein on the surface of moDCs did not cause any defect in the engulfment of *Escherichia coli (E. coli)* cells [22]. Similarly, we did not observe any significant differences between the internalizing capacity of CD1a^−^ and CD1a^+^ moDCs activated with WT or ∆Lc-p75 mutant *L. casei* BL23 (Figure 6A,B).

These observations indicated that the *L. casei* PGH’s targeted mutation modified the fate of the bacteria through the inhibition of their efficient uptake by phagocytic moDCs. This phenomenon may have been the consequence of the chain-forming property of the mutant bacterial strain. The absence or presence of the cell surface CD1a molecule did not influence the phagocytosis of *L. casei* strains by moDCs.

### 2.6. Inhibition of Phagocytosis Diminishes the Effects Triggered by WT L. casei BL23 on moDC Functions

**Figure 7:**
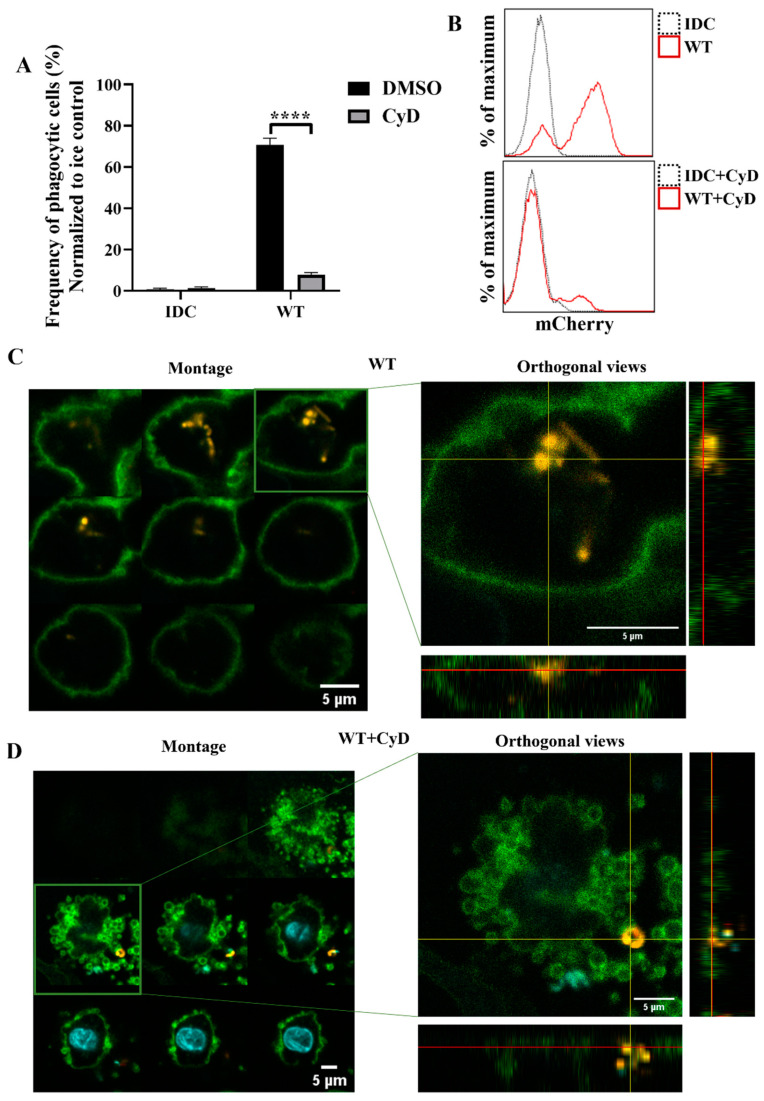
Inhibition of the cytoskeleton rearrangement causes impaired phagocytosis of wild-type *L. casei* BL23 by moDCs. Five-day moDCs were preincubated with 15 μM cytochalasin D (CyD) or the vehicle control DMSO for 30 min. Then, moDCs were activated with four times more mCherry-expressing wild-type bacteria for 24 h. Phagocytosis of the WT bacteria by moDCs was detected by flow cytometry and confocal microscopy. The frequency of the phagocytic moDCs at 37 °C was normalized to ice control and was calculated from 3 independent experiments ±SD (**A**). IDC means the nonactivated moDC. Histogram overlays show one representative experiment (**B**). For the confocal microscopic pictures (**C**,**D**) the moDCs are labeled with HLA-DQ (green) and DAPI (cyan); mCherry is indicated with yellow. On the left of the figure, montage pictures show the Z-stack images per 1 µm from the bottom to the top of the cell. On the right of the figure, the orthogonal views of one Z-stack image are presented. The bottom shows the Z-projection in the X–Z direction; on the right is the Z-projection in the Y–Z direction. The red lines indicate the Z-depth of the optical slice and the orthogonal planes of the X–Z and Y–Z projections, respectively. Two-way ANOVA followed by Tukey’s multiple-comparison tests were used in the statistical analysis. The difference between the nontreated and CyD-treated moDCs was statistically significant and defined as **** *p* < 0.0001.

To further investigate whether phagocytosis was the essential step in activating moDCs by WT *L. casei* BL23, we inhibited actin polymerization, the early steps of phagocytosis, using cytochalasin D (CyD). Firstly, we determined the phagocytic activity of moDCs using flow cytometry and confocal microscopy. The CyD treatment induced a significant decrease in the WT bacteria’s engulfment by moDCs compared to the nontreated moDCs (Figure 7A,B). To check that impairment of the phagocytosis in WT bacteria was not the consequence of increased cell death caused by CyD, we measured the frequency of the dead DCs using 7AAD staining. Importantly, we could not detect any significant cell death in the CyD-treated and nontreated samples after coincubation with WT *L. casei* BL23 (Figure S4A). Confocal microscopic montage pictures and the orthogonal views revealed that moDCs phagocytosed the WT bacteria after 24 h (Figure 7C). However, after the CyD preincubation, mCherry-positive bacteria were attached only to the cell surface of moDCs (Figure 7D).

**Figure 8:**
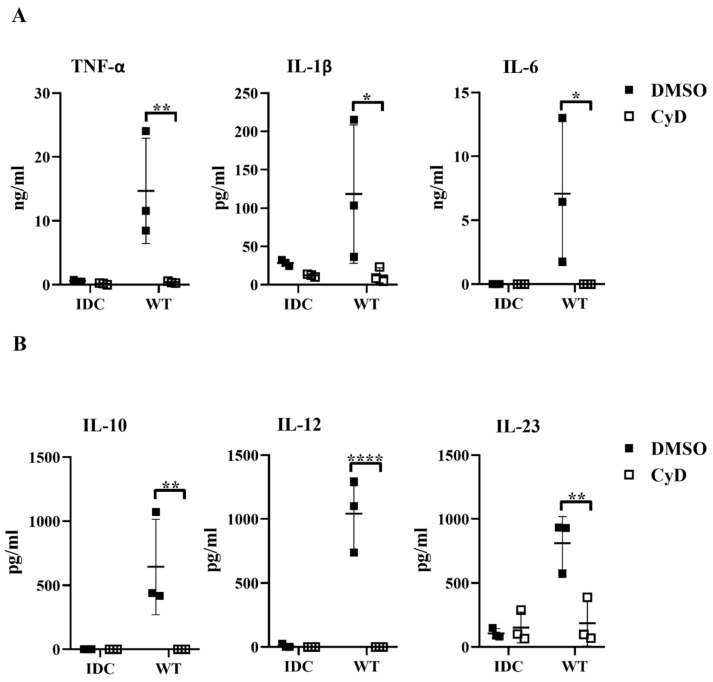
Blocked phagocytosis leads to reduced cytokine secretion by bacteria-exposed moDCs. Five-day moDCs were preincubated with 15 μM CyD or the vehicle control DMSO for 30 min. Then, moDCs were stimulated with WT *L. casei* at a ratio of 1:4 for 24 h. IDC means cells without bacterial treatment. The concentrations of proinflammatory TNF-α, IL-1β, and IL-6 cytokines (**A**) and the T-cell-polarizing IL-10, IL-12, and IL-23 (**B**) were determined from the supernatants of the WT-bacteria-activated moDCs and control IDCs. Figures represent the mean of 3 independent donors ±SD. Each dot represents one donor. The Student’s paired two-tailed *t*-test was used in the statistical analysis, with significance defined as * *p* < 0.05, ** *p* < 0.01, and **** *p* < 0.0001 between the CyD- and DMSO-treated moDCs.

**Figure 9:**
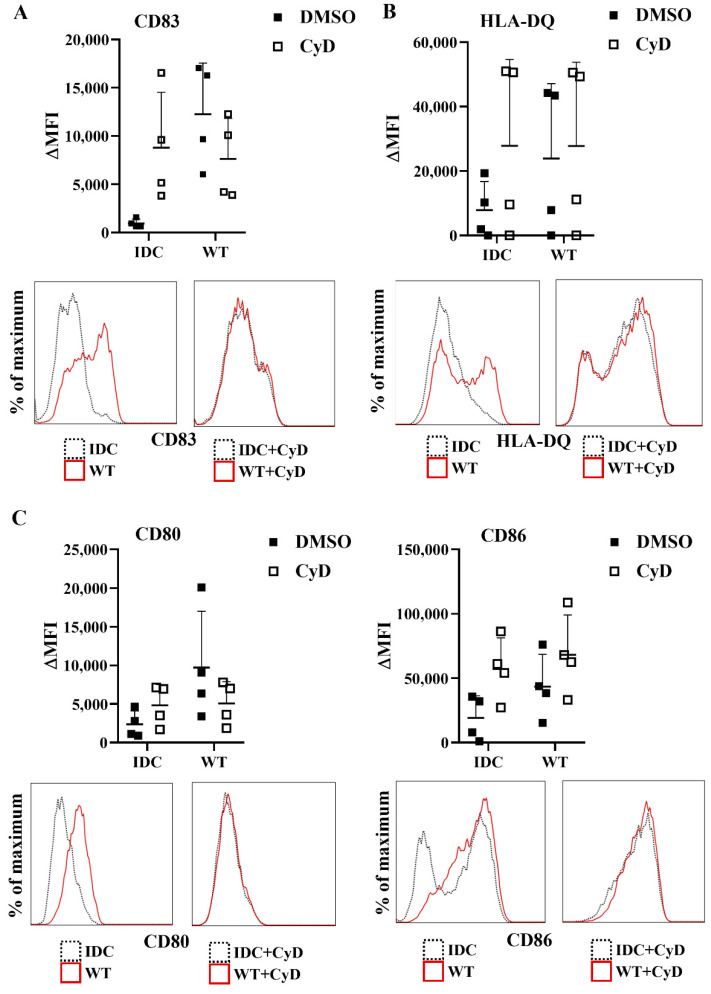
Cytochalasin D pretreatment does not cause alterations in the expression of activation and costimulatory markers of moDCs. Five-day moDCs were preincubated with 15 μM CyD or its vehicle control DMSO for 30 min. Then, moDCs were stimulated with WT *L. casei* BL23 at a ratio of 1:4 for 24 h. Expression of CD83 (**A**), HLA-DQ (**B**), and costimulatory CD80 and CD86 (**C**) molecules was measured by flow cytometry. IDC represents cells without any bacterial treatment. Median fluorescent intensities (ΔMFI) were calculated from 4 independent experiments ±SD. Histogram overlays show one representative experiment. Each dot is representative of one donor. Two-way ANOVA followed by Tukey’s multiple-comparison tests was used in the statistical analysis.

Next, we tested whether the inhibition of the phagocytosis had a blocking effect on cytokine secretion by human moDCs. We found that impairment of the bacterial engulfment caused a highly significant decrease in proinflammatory TNF-α, IL-1β, and IL-6 cytokine secretion (Figure 8A). Furthermore, CyD treatment attenuated the IL-10, IL-12, and IL-23 T-cell-polarizing cytokine release by *L. casei*-activated moDCs (Figure 8B), and consequently reduced the number of IFNγ- and IL-17A-producing T cells in the DC–T cell cocultures (Figure S4B). It should be noted that CyD treatment induced significantly increased IL-8 chemokine production by moDCs even in the absence of bacteria (Figure S4C), which was in good agreement with previous results obtained with human retinal pigment epithelial cells [23]. Importantly, CyD pretreatment did not influence the cell surface expression of CD83, HLA-DQ (Figure 9A,B), and the costimulatory CD80 and CD86 molecules (Figure 9C).

Overall, these results demonstrated that bacteria internalization is an essential step in *L. casei* BL23-induced moDC activation, indicating that the reduced phagocytic uptake of ∆Lc-p75 mutant *L. casei* BL23 may result in its decreased moDC-activating capacity.

## 3. Discussion

As a mucosal surface, the gastrointestinal tract is a typical entry of pathogens. However, on the other hand, the gut harbors a large number of commensal microorganisms, such as LAB. LAB exerts various beneficial effects on the human host; hence, an essential role of the gut immune system is to distinguish the pathogen-derived antigens from those derived from the microbiota. Due to their ability to precisely sense the local microenvironment, DCs can control both the immunogenic and tolerogenic responses according to the features of the engulfed antigens [24]. DCs can directly [25] or indirectly [26] sample the luminal content of the gut, such as bacteria or their derived components and metabolites. Furthermore, the unique, naïve T-cell-activating capacity of DCs is essential in inducing tolerance or adaptive immune responses against the microbiota members. Therefore, the interaction between LAB members such as lactobacilli and DCs is under extensive investigation. In this study, we investigated the effects of PG alterations in the cell wall of *L. casei* BL23 on moDCs’ activation and their T-cell-polarizing ability.

Our findings demonstrated that the ΔLc-p75 mutant *L. casei* strain induced efficient maturation of moDCs while also causing dampened cytokine secretion by these cells. This result was in line a previous observation showing that potentially probiotic bacteria induced expression of moDC maturation and activation markers as efficiently as pathogenic bacteria, but their ability to trigger cytokine production varied significantly from one bacterial strain to another [27]. Among the investigated proinflammatory soluble mediators, IL-8 was an exception, as we could not detect any significant differences in its secretion by moDCs activated with the WT or PGH mutant *L. casei* strains. This observation suggested that the gene expression of proinflammatory cytokines and IL-8 was differentially regulated in myeloid cells. Indeed, analysis of recruitment of p65 NF-kB to the corresponding promoters in U937 macrophages revealed that although the p65 recruitment to TNF-α, IL-1β, and IL-6 promoters was inhibited by the nuclear IkBα, recruitment to IL-8 promoter was not repressed. Furthermore, the IL-8 promoter was specifically associated with the S536-phosphorylated form of p65 NF-kB, whereas only the unphosphorylated p65 was recruited to promoters of TNF-α, IL-1β, and IL-6 genes [28]. Another study reported that knockdown of regulator of G-protein signaling 16 (RGS16) by RNAi in the human promonocytic cell line THP-1 upregulated TNF-α, IL-1β, and IL-6, but not IL-8 [29]. Our finding that the Lc-p75-defective *L. casei* induced reduced Th1 and Th17 responses compared to WT bacteria was not surprising, given our observation that mutant bacteria triggered lower production of IL-12 and IL-23, which are major cytokines required for polarization and maintenance of the Th1 and Th17 subsets, respectively [30], by moDCs compared to their WT counterpart.

To determine whether the observed differences between WT and mutant *L. casei* strains in DC activation were the direct consequences of the modified bacterial cell wall constituents, we tested the moDC-stimulating ability of purified PGs from the WT and ΔLc-p75 mutant strains. We found that the purified PGs from WT and mutant *L. casei* BL23 strains had a similar immunomodulatory effect on moDCs. It has long been known that PGs can be recognized by PRRs in the host cells and mediate their activation. In 1999, it was demonstrated that their recognition was TLR2-mediated [31]. However, soon after, Travassos et al. found that the intracellular Nod proteins were more potent candidates in the sensing of Gram-positive bacterial PGs [32]. Therefore, PGs or their derived fragments had to enter the intracellular space of the host cell. Various mechanisms help the uptake of PGs, such as phagocytosis, transporter-mediated translocation, and micropinocytosis-like mechanisms [33], or the action of bacterial PGHs, which can generate PG fragments [34]. Based on our results, no matter how they entered the moDCs, purified PG components from the ΔLc-p75 mutant or WT bacteria activated their receptors with the same efficiency.

Several studies demonstrated that DCs could phagocytose different *Lactobacillus* strains, which induced distinct DC activation patterns. A recent study showed that human moDCs were able to uptake *L. rhamnosus* JB-1 bacteria after 48 h, which caused poor costimulatory molecule expression and weak cytokine production [35]. In another publication, mucus-adhesin-expressing *L. reuteri* strains were efficiently phagocytosed by moDCs [19]. In agreement with these data, our phagocytosis results revealed that human moDCs were able to uptake *L. casei* BL23. Moreover, our results showed that the long-chain-forming mutant bacteria were weakly internalized by moDCs.

The regulation of the phagocytic process is multifaceted, and is determined by the involved receptors, duration of the binding, and the physical characteristics, such as the size of the engulfed material [36]. It was previously shown that bacterial aggregates were weakly phagocytosed by polymorphonuclear leukocytes (PMN), influencing the killing ability of PMNs [37]. Thus, the properties of phagocytosis may consequently determine the nature of developing responses. Indeed, it has been illustrated that in addition to the signaling initiated by TLR2 receptor, bacterial phagocytosis and Nod proteins were also important for macrophage responsiveness to Gram-positive bacteria [38]. Our results showed that the long-chain-forming mutant of *L. casei* BL23 could induce only a weak pro-inflammatory and T-cell-polarizing cytokine secretion by human moDCs as compared to WT bacteria. The importance of phagocytosis in cytokine production was further confirmed by its inhibition with cytochalasin D, which strongly reduced the concentration of secreted proinflammatory and T-cell-polarizing cytokines. The observed phenomenon was similar to the case when moDCs were stimulated with the long-chain-forming ΔLc-p75 mutant bacteria.

It has been demonstrated that there was a Nod2-mediated enhancement of TLR-induced TNF-α, IL-12, and IL-10 production in DCs [39]. Nod2fs mutation, detected in patients suffering from Crohn’s disease, results in a loss-of-function phenotype in human myeloid DCs. Dendritic cells derived from Crohn’s disease patients homozygous for this mutation respond normally to purified TLR ligands but fail to upregulate cytokine production in response to treatment with a Nod2 ligand [39]. We assumed that a similar phenomenon was taking place in moDCs exposed to the long-chain-forming ΔLc-p75 mutant bacteria. Signals triggered by TLR2 on the cell surface induced the production of proinflammatory and T-cell-polarizing cytokines, but this remained at a relative low level due to the lack of Nod2-mediated enhancement resulting from the less efficient uptake of mutant bacteria. However, this hypothesis needs further investigation.

Taken together, our results indicated that the immunomodulatory properties of *L. casei* BL23 bacteria were highly dependent on their phagocytosis by human moDCs. Internalization determined the cytokine secretion of moDCs, which overall could be translated to inflammation and also to Th1/Th17-mediated adaptive immune responses. The long-chain-forming ΔLc-p75 mutant *L. casei*, which are less likely to be phagocytized, induced only moderate DC and T cell responses. Our findings may be useful in the generation of new probiotic agents.

## 4. Materials and Methods

### 4.1. Bacterial Strains and Growth Conditions

*L. casei* BL23 [40] and its ∆Lc-p75 mutant derivative obtained by the deletion of the *lcabl_02770* gene encoding the Lc-p75 PGH [15] were used in this study. They were inoculated from frozen glycerol stocks on MRS agar plates and cultivated at 37 °C for 48 h to obtain isolated colonies. Single colonies were diluted in MRS broth (BD Difco, Fisher Scientific, Co., L.L.C., Pittsburgh, PA, USA) and propagated for 16 h at 37 °C until the beginning of the stationary phase. Fluorescent mCherry strains were obtained by transformation with a plasmid-encoding red fluorescent mCherry protein (pTS-mCherry; kind gift of Jerry M. Wells, Wageningen University, Wageningen, The Netherlands). The red fluorescent strains were cultured with 5 μg/mL erythromycin (Merck KGaA, Darmstadt, Germany) included in the MRS medium to select the plasmid. An optical density (OD) of 1 at 600 nm corresponded to 2.5 × 10^8^ cells per ml for the wild-type *L. casei* BL23 strain, as calculated from a dilution series of liquid bacterial culture and colony counting.

To verify that the same OD values for cell suspensions of the long-chain-forming mutant or wild-type strain corresponded to similar numbers of bacterial cells, bacteria were cultivated for 16 h, then stained in suspension at OD_600nm_ with 4′,6-diamidino-2-phenylindole (DAPI), a fluorescent DNA dye, and the bacteria-associated fluorescence was measured. We observed that for both strains under study, the OD_600nm_ value was perfectly correlated with the fluorescence intensity level, and thus with the cell number (Figure S5).

### 4.2. Extraction of Peptidoglycan

PG was extracted from *L. casei* strains as described previously [15] with some modifications. Cells from a 500 mL exponentially growing culture (OD_600_, 0.3) were chilled on ice and harvested using centrifugation. Cells were suspended in deionized H_2_O and boiled for 10 min. They were then resuspended in 5% (*w*/*v*) SDS in 50 mM Tris-HCl pH 7.0 and boiled for 25 min. The pellet obtained by centrifugation at 20,000× *g* for 10 min was resuspended in 4% (*w*/*v*) SDS in Tris-HCl buffer and boiled again for 15 min. Cell walls were recovered using centrifugation at 20,000× *g* for 10 min and washed six times with deionized H_2_O to remove SDS. The cell wall pellet was then successively treated with Pronase (2 mg/mL) for 90 min at 60 °C, with α-amylase (50 µg/mL) for 2 h at 37 °C, with DNase (50 µg/mL) and RNase (50 µg/mL) for 4 h at 37 °C, and lipase (50 µg/mL) and finally by trypsin (200 µg/mL) for 16 h at 37 °C. The final pellet was then treated with 2% SDS in Tris-HCl (50 mM pH 7.0.) and washed with deionized H_2_O. The insoluble pellet was treated with 48% fluorhydric acid overnight at 4 °C to remove wall polysaccharides (WPS) [41]. After centrifugation, the pellet (containing PG) was washed several times with 0.25 M Tris-HCl, pH 7.0 and deionized H_2_O. The final pellet was lyophilized and stored at −20 °C.

### 4.3. Human Monocyte Separation and Differentiation to Dendritic Cells

Peripheral blood mononuclear cells (PBMCs) were isolated from heparinized leukocyte-enriched buffy coats by applying a Ficoll-Paque Plus (Amersham Biosciences, Uppsala, Sweden) density gradient centrifugation. Monocytes were separated from PBMCs with magnetic cell separation using CD14-specific antibody-coated microbeads, according to the manufacturer’s instructions (MiltenyiBiotec, Bergisch Gladbach, Germany). After separation on a VarioMACS magnet, the homogeneity of the isolated CD14^+^ monocyte fraction was greater in all experiments than 90–95% as checked by flow cytometry. The autologous monocyte-depleted PBL fraction was used in the ELISPOT assays as a T cell source.

Isolated monocytes were seeded at 1 × 10^6^/mL concentration in serum-free AIM-V medium (Thermo Fisher Scientific, Waltham, MA, USA) supplemented with 100 ng/mL IL-4 (PeproTech EC, London, UK) and 80 ng/mL GM-CSF (Gentaur Molecular Products, Brussels, Belgium), and were differentiated to moDCs for 5 days. On day 2, half of the medium was changed and complemented with 100 ng/mL IL-4 and 80 ng/mL GM-CSF. When indicated, moDCs were preincubated with 15 µM cytochalasin D (CyD, Merck KGaA) dissolved in dimethyl sulfoxide (DMSO, Serva Electrophoresis GMBH, Heidelberg, Germany) for 30 min to inhibit the phagocytosis. DMSO was used as a vehicle control.

### 4.4. Stimulation of moDCs with Bacteria or Their PG Fragments

Bacteria were washed twice with cold PBS and added to the moDCs on day 5 of in vitro differentiation at a ratio of 1 (moDC):4 (bacteria) for 3 or 24 h. Control cells were left untreated (IDC).

Purified bacterial PG samples were resuspended in deionized water and sonicated with Branson Sonifier 450 until they became clear. MoDCs were treated with 10 µg/mL PG fragments for 24 h.

Each experiment was repeated with at least three independent donors.

### 4.5. Analysis of the Expression of Cell Surface Markers and Cell Viability of Dendritic Cells by Flow Cytometry

WT and Lc-p75 mutant Lactobacilli were washed with PBS and added to the five-day moDCs for 24 h at a ratio of 1:4. In parallel, purified lactobacterial PG fragments were resuspended in deionized water and sonicated. MoDCs were treated with 10 µg/mL PG fragments for 24 h. On day 6, cells were stained with fluorescence-conjugated monoclonal antibodies: CD83-fluorescein isothiocyanate (FITC), CD80-FITC, CD86-phycoerythrin (PE), and HLA-DQ-FITC (BioLegend, San Diego, CA, USA) or left unlabeled as a control. Nonspecific antibody binding was inhibited using heat-inactivated mouse serum. The moDC population was gated according to the forward/side scatter parameters. Fluorescence intensities were measured using a ACEA NovoCyte 2000R cytometer (Agilent, Santa Clara, CA, USA); data analyses were performed with the FlowJo vX.0.7 software. Viable and nonviable cells were dissected using flow cytometry after staining the freshly collected, nonfixed cells with 0.5 µg/mL 7-amino-actinomycin D (7-AAD) (Merck KGaA, Darmstadt, Germany).

### 4.6. Cytokine Measurements by Enzyme-Linked Immunosorbent Assays (ELISA)

Supernatants of stimulated moDCs were collected after 24 h, and the concentrations of IL-1β, IL-6, TNF-α, IL-12p70, IL-10, IL-23, and IL-8 were measured by using BD OptEIA ELISA kits according to the manufacturer’s instructions (Becton Dickinson, BD Biosciences, Franklin Lakes, NJ, USA). OD was detected at 450 nm using a Synergy^TM^ HT Multi-Detection Microplate reader (Bio-Tek Instruments, VT, USA) and KC4 software v3.4.

### 4.7. ELISPOT Assays

Bacteria-stimulated moDCs were counted, washed with PBS, and cocultured with autologous PBL at a ratio of 1:20 in serum-free AIM-V medium for 3 days at 37 °C in a 5% CO_2_ atmosphere. On day 4, the cells were collected, counted, and subjected to IFNγ, IL-17A, and IL-4 Ready Set Go ELISPOT assays according to the manufacturer’s instructions (eBioscience, San Diego, CA, USA). Briefly, 300,000 cells/well were incubated in CTL-Test medium (Cellular Technology Limited, Cleveland, OH, USA) for 40–48 h at 37 °C in MultiScreen-HTS PVDF plates (Millipore S.A., Molsheim, France) precoated with capture antibodies specific for IFNγ, IL-4, or IL-17A. Together with the IL-4- and IL-17A-specific capture antibodies, 0.5 µg/mL purified antihuman CD3 antibody (BD Biosciences) was added to the coating buffer for the mitogenic stimulation of CD3^+^ T cells. The detection of the cytokine release was performed using biotinylated IFNγ-, IL-4-, or IL-17A-specific antibodies in the presence of HRP conjugated to avidin. Soon after, the colorigenic substrate, 3-amino-9-ethylcarbazole (AEC Substrate Set, BD Biosciences) was added. The color development was stopped by tap water, and air-dried plates were analyzed with a computer-assisted ELISPOT image analyzer (Series 1 ImmunoSpot Analyzer, ImmunoSpot Version 4.0 Software Academic, Cellular Technology Limited, Shaker Heights, OH, USA).

### 4.8. Phagocytosis Assay by Flow Cytometry

The moDCs and mCherry-expressing bacteria were coincubated at a ratio of 1:4 for 3 or 24 h at 37 °C in a 5% CO_2_ atmosphere or on ice as a control. After the incubation period, moDCs were stained with allophycocyanin (APC)-labeled anti-CD1a mAb (BioLegend, San Diego, CA, USA). Cell analysis was performed with an ACEA NovoCyte 3000 RYB flow cytometer (Agilent). Data analyses were performed with the FlowJo vX.0.7 software (Tree Star Inc., Ashland, OR, USA). The moDC population was gated according to the forward/side scatter parameters by excluding the noninternalized, free bacteria. The phagocytosis ratio was determined as a percentage of mCherry-positive cells or according to the median values of mCherry fluorescence intensity (MFI). Similarly, the frequency of phagocytic cells was determined in the CD1a-positive and the CD1a-negative moDC subpopulations as a percentage of mCherry-positive cells.

### 4.9. Confocal Microscopy

A Zeiss LSM 880 confocal laser scanning microscope (Carl Zeiss, Oberkochen, Germany) equipped with 40×x water-immersion objective (NA 1.2) was used to image the phagocytosis of *L. casei* cells by moDCs after 24 h. For the excitation of DAPI, a 405 nm diode laser was used; for FITC, the 488 nm line of an Argon ion laser was used; for mCherry, a 543 nm He-Ne laser was used; and for APC, a 633 nm He-Ne laser was applied. Fluorescence emissions of DAPI and FITC were detected in the wavelength ranges of 410–485 and 490–610 nm, respectively, while the detection of mCherry was performed with a 575–695 nm bandpass filter. For the detection of APC, a 640–745 nm bandpass filter was used. Z-stack images were collected at 1 µm intervals from the bottom to the top of the cells.

### 4.10. Statistical Analysis

Analyses were performed using Microsoft Excel and GraphPad Prism v8.0 (GraphPad Software Inc., San Diego, CA, USA). Comparison between two groups was performed using a paired, two-tailed Student’s *t*-test. One-way ANOVA followed by Tukey’s post hoc test was used for the comparisons of more than 2 groups. Two-way ANOVA followed by Tukey’s post hoc test was applied for the comparisons of two independent variables. The results are shown as mean ± standard deviation (SD). Differences were considered to be statistically significant at *p* < 0.05. Significance was indicated as * *p* < 0.05, ** *p* < 0.01, *** *p* < 0.001, and **** *p* < 0.0001; or as # *p* < 0.05, ## *p* < 0.01, ### *p* < 0.001, or #### *p* < 0.0001.

## Data Availability

Not applicable.

## Supplementary Materials

The following supporting information can be downloaded at: https://www.mdpi.com/article/10.3390/ijms23147620/s1.
