# Supplementary material for: The Phagocytosis of Lacticaseibacillus casei and Its Immunomodulatory Properties on Human Monocyte-Derived Dendritic Cells Depend on the Expression of Lc-p75, a Bacterial Peptidoglycan Hydrolase
Source: Int J Mol Sci. 2022 Jul 10;23(14):7620. doi: 10.3390/ijms23147620 (PMC9319067; doi:10.3390/ijms23147620)
Supplement: Supplementary file 1 [file ijms-23-07620-s001.zip › ijms-1764406-supplementary.pdf]

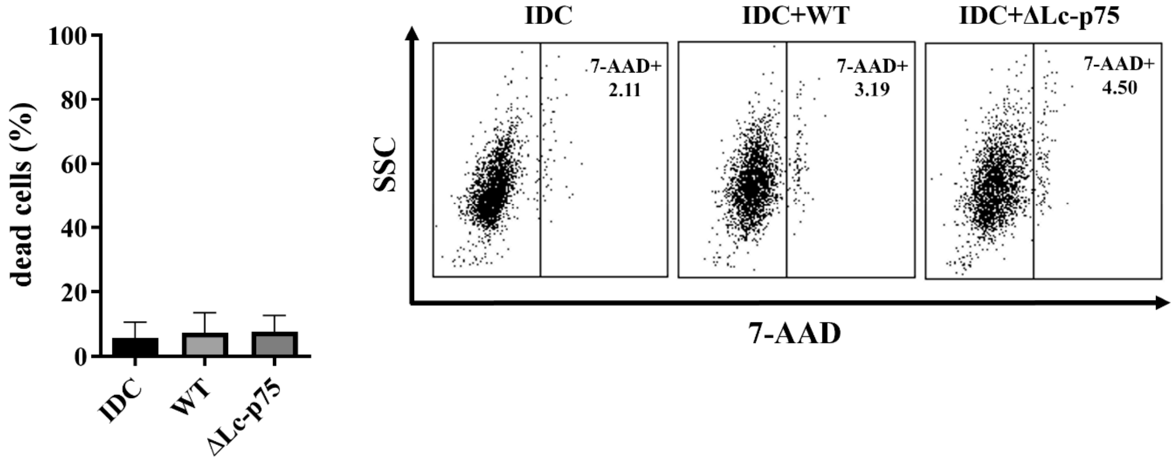

Figure S1. Activation with *L. casei* strains or derived PGs derived from them does not alter the viability of the moDCs. In-vitro differentiated five-day moDCs were stimulated with WT *L. casei* BL23 and its PGH mutant at a moDC: bacteria ratio of 1:4 at 37°C for 24 h. Negative controls included moDCs incubated without bacteria (IDC). The percentage of dead cells was determined by 7-AAD fluorescent intercalator dye using flow cytometry. Mean values were calculated from 4-6 independent experiments ±SD. Student's paired two-tailed *t*-test was used in the statistical analysis.

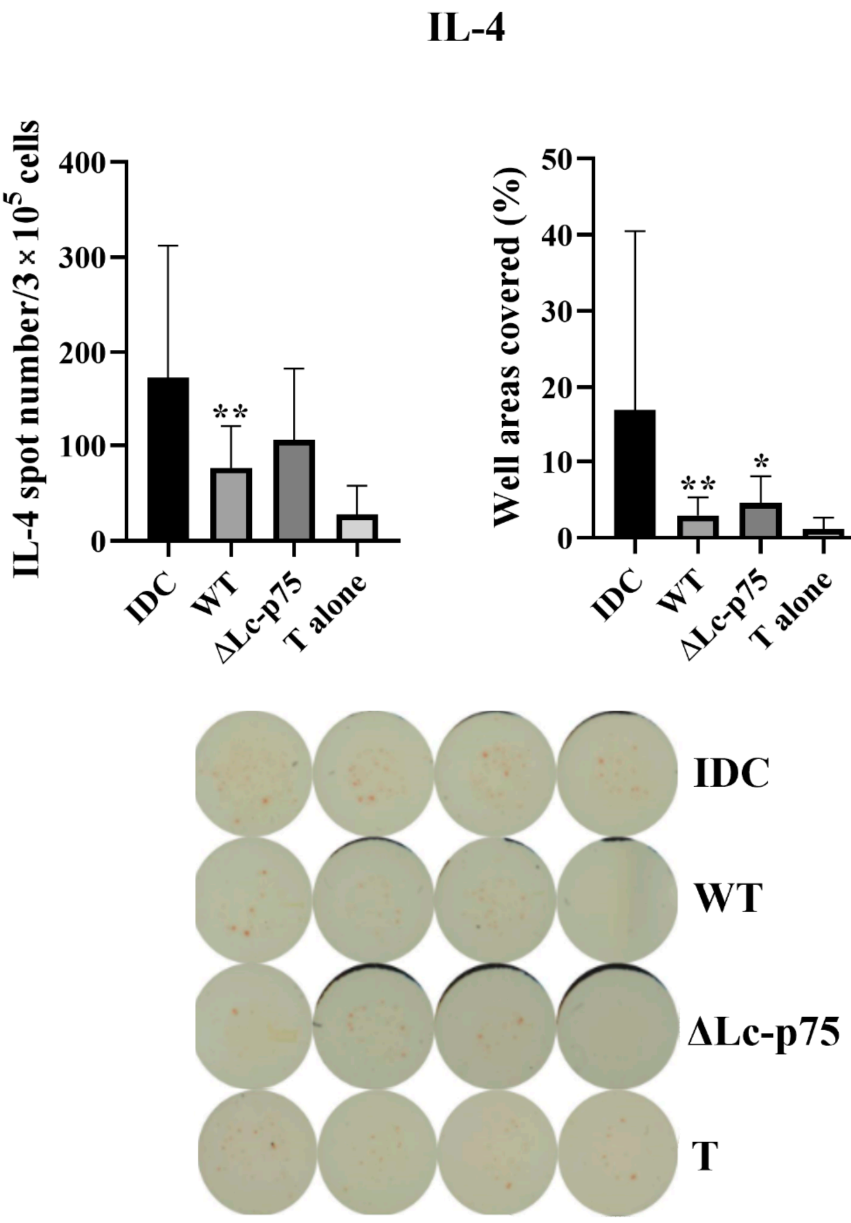

Figure S2. Exposure to *L. casei* BL23 reduces the number of IL-4-producing T cells primed by moDCs. 5-day moDCs activated with wild-type and Lc-p75 mutant *L. casei* BL23 were washed and co-cultured with autologous T cells from freshly isolated PBL at a ratio of 1:20 for 3 days. The frequency of cytokine-producing T lymphocytes was measured by IL-4 ELISPOT assay. The spot number was counted, or the area covered by the spots was calculated by a computer-assisted ELISPOT image-analyzer. The mean value of spot numbers and well areas covered were calculated from 5 independent experiments with 4-6 parallel wells ±SD. One-way ANOVA followed by Tukey's multiple comparison test was used for statistical analysis. Significance defined as * $p < 0.05$ and ** $p < 0.01$ compared to control, non-treated samples (IDC).

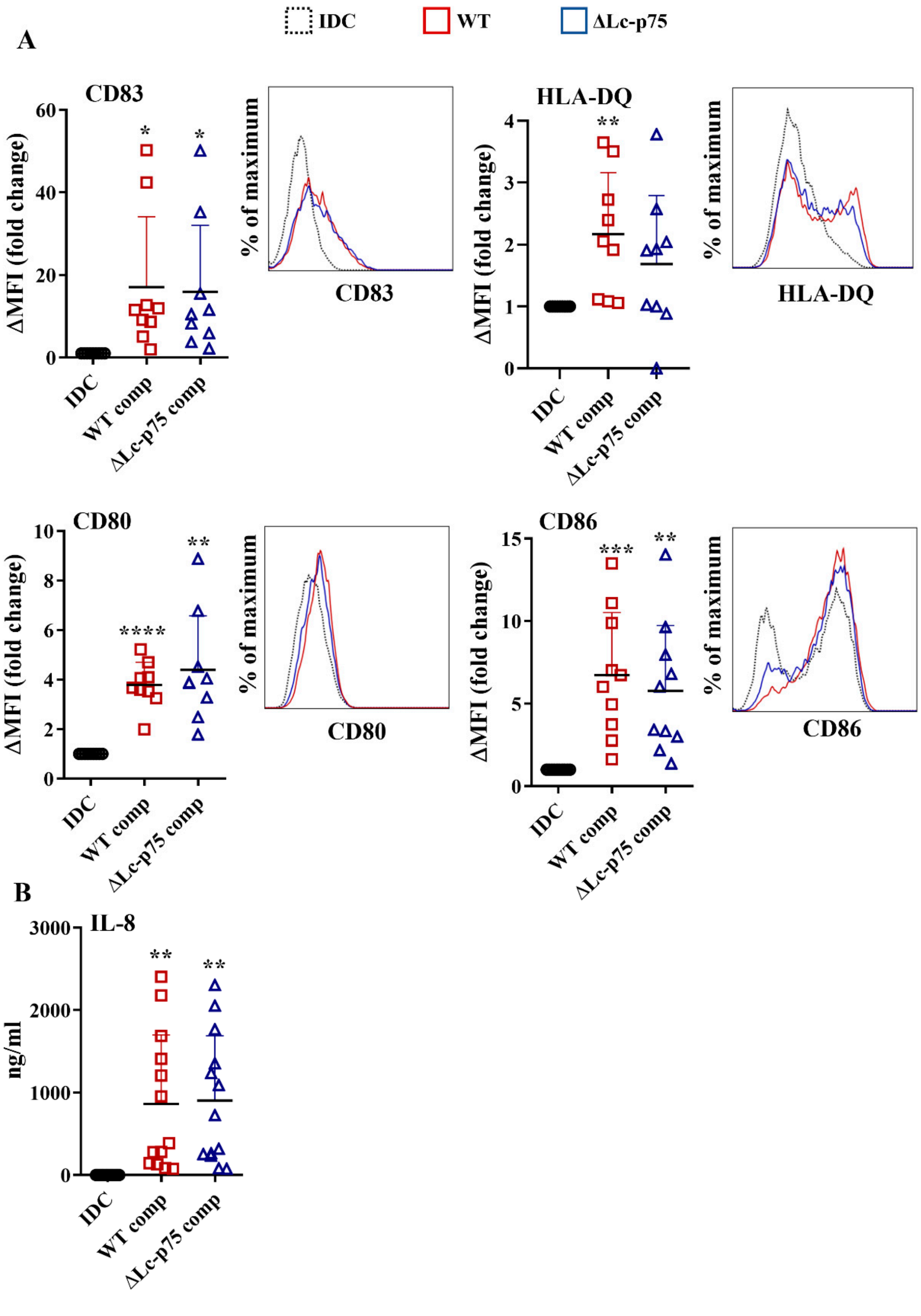

Figure S3. PGs derived from *L. casei* BL23 WT and mutant bacteria induce the expression of CD83, HLA-DQ, CD80, and CD86 and IL-8 secretion by moDCs at similar levels. 5-day moDCs were activated with 10 µg/ml PG fragments from WT and Lc-p75 mutant bacteria. Expression of CD83, HLA-DQ, CD80 and CD86 (A) was measured by flow cytometry. Fold change of median fluorescent intensities (ΔMFI) to the control, non-treated cells (IDC) was calculated from 7-9 independent experiments ±SD. The histograms show one representative experiment. Concentration of IL-8 chemokine (B) was determined by ELISA from 12 independent donors. The figure represents the mean ±SD. Each dot is representative for one donor. Student's paired two-tailed *t*-test was used in the statistical analysis. Significance defined as * p <0.05, ** p <0.01, and **** p <0.0001 compared to control samples (IDC).

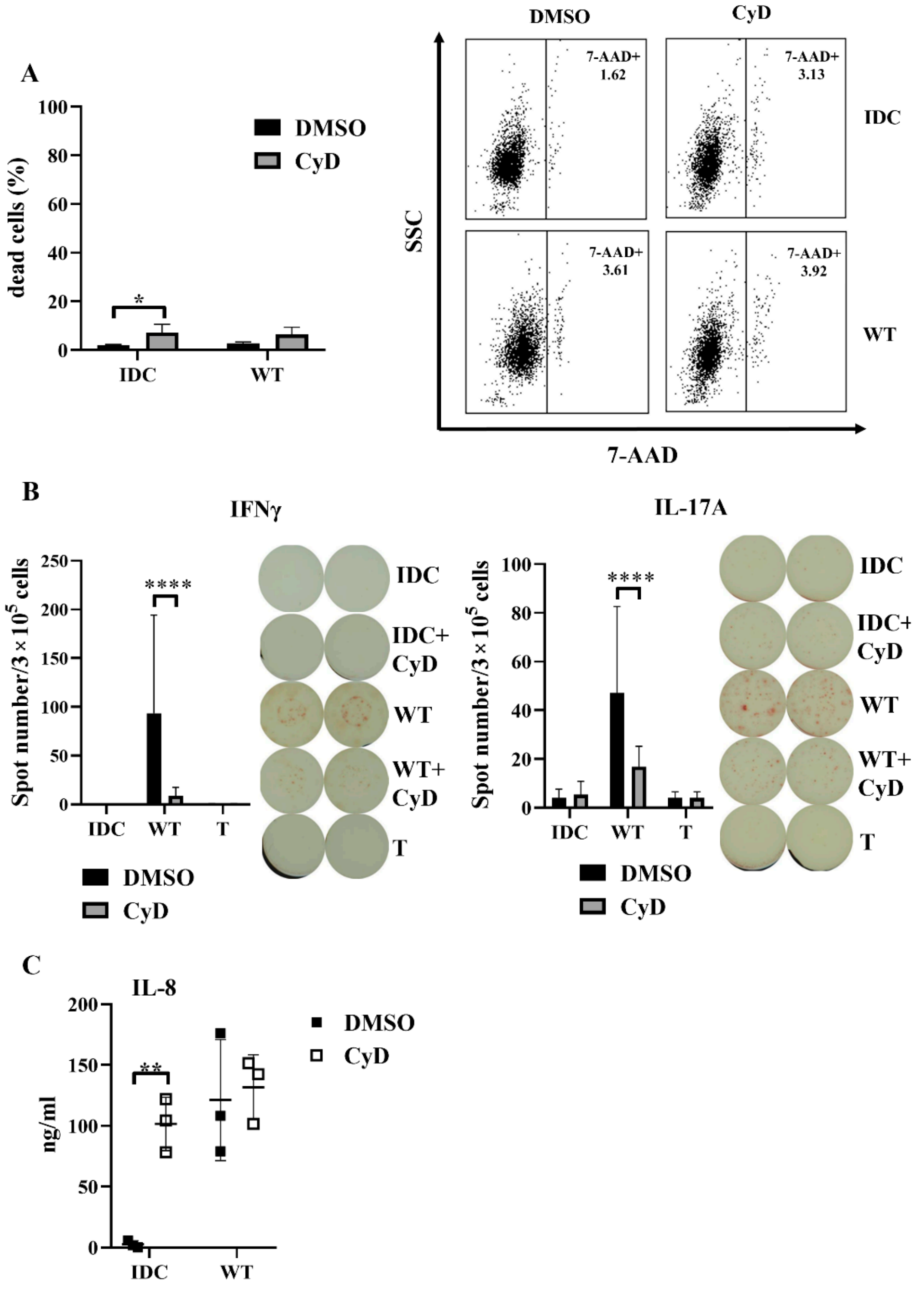

Figure S4. CyD treatment does not increase the death of moDC but decreases the number of IFN-γ and IL-17A producing T cells after *L. casei* activation and elevates the IL-8 production by moDCs regardless of bacterial presence. 5-day moDCs were preincubated with 15 μM CyD or its vehicle control DMSO for 30 min. Then, moDCs were stimulated with wild-type *L. casei* BL23 at a ratio of 1:4 for 24 h. IDC means moDC without any bacterial activation. The percentage of dead cells was determined by 7-AAD fluorescent intercalator dye using flow cytometry (A). 5-day moDCs were pretreated 15 μM CyD or the vehicle control DMSO for 30 min followed by activation with four times more wild-type *L. casei*. After 24 h moDCs were washed and co-cultured with autologous T cells from freshly isolated PBL at a ratio of 1:20 for 3 days. The frequency of cytokine-producing T lymphocytes was measured by IFN-γ or IL-17A (B) ELISPOT assays. The spot number was counted by a computer-assisted ELISPOT image-analyzer. The mean value of spot numbers was calculated from 3 independent experiments with 4 wells ±SD. The concentration of IL-8 (C) was determined from the supernatants of the moDCs. Each dot represents one donor. Mean values were calculated from 3 independent experiments ±SD. Two-way ANOVA followed by

Tukey's multiple comparison test was used for statistical analysis. Significance defined as * p <0.05 and ** p <0.01, **** p <0.0001 compared to DMSO-treated moDCs.

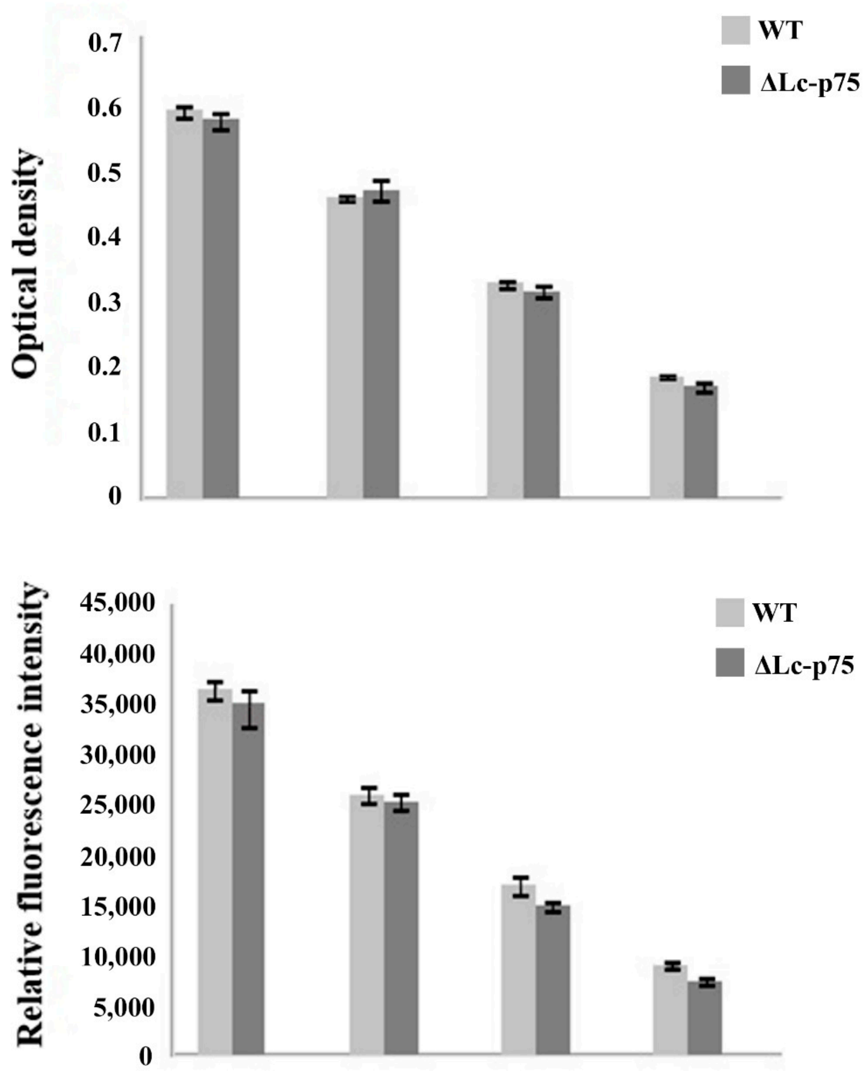

Figure S5. Correspondence between OD_{600nm} values of bacteria suspension and number of bacteria as measured by DAPI staining for *L. casei* BL23 and its derivative ΔLc-p75 mutant. OD_{600} values (A) and relative fluorescence intensity (B) after DAPI staining were measured at the same time (after 16 h) for each sample (numbered 1 to 4) and strain, respectively, with a Tecan Infinite M200 multimode microplate reader.
